# Impact of COVID-19 pandemic on senior-to-senior social engagement activities at a community space in Japan

**DOI:** 10.1093/geroni/igaa057.3473

**Published:** 2020-12-16

**Authors:** Neo Kazembe, Hongjik Kim, Cong Wang, Shuichiro Higuma, Yerim Yang, Taeeun Kim, Ryogo Ogino, Mai Takase

**Affiliations:** 1 The University of Tokyo, Tokyo, Japan; 2 Saga University, Saga, Japan

## Abstract

A local community space in Japan, Chiki Kastudokan, has been hosting several senior-to-senior social activities for community-dwelling older adults with support from the University of Tokyo. The activities are aimed at fostering communication and social engagement through exercise, music, cafe, and craft. After the emergence of COVID-19 pandemic, these activities have been disrupted. We conducted a questionnaire survey between June and July 2020 to assess how the pandemic has affected 26 activities and identify support needs that can enable their continuity amid the pandemic (response rate: 77%). First, all 26 activities were suspended by the onset of COVID-19. Hosts of 8 (40%) activities devised alternative ways to engage participants at home. For instance, hosts of craft activities arranged and sent to participants crafting kits. Limited capacity in using online platforms like Skype or Zoom prevented virtual interaction of hosts and participants. Notably, hosts were anxious towards resuming activities amid the pandemic. They worried about their health (50%), of being criticized by friends and family(25%), and of infections spreading among participants (85%). Hosts wanted strategies to prevent infections during activities, and easy to understand infection prevention guidelines for participants. These findings reveal that COVID-19 pandemic has negatively affected senior-to-senior activities at Chiki Kastudokan. The University of Tokyo could help hosts identify effective infection prevention strategies to use when hosting activities amid the pandemic. It could also provide ICT training to hosts and participants to enhance their capacity in using online platforms in case of future waves of COVID-19.

